# Supplementary material for: Synthesis, characterization, and in vivo safety evaluation of propylated Dioscorea abyssinica starch
Source: PLoS One. 2022 Nov 28;17(11):e0276965. doi: 10.1371/journal.pone.0276965 (PMC9704604; doi:10.1371/journal.pone.0276965)
Supplement: S4 Fig — (DOCX) [file pone.0276965.s004.docx]

| Temperature (^0^C) | NDAS(DS=00)  (X±SD) | | PDAS(DS=0.453)  (X±SD) | | PDAS(DS=0.474)  (X±SD) | | PDAS(DS=1.710)  (X±SD) | | PDAS(DS=2.842)  (X±SD) | |
| --- | --- | --- | --- | --- | --- | --- | --- | --- | --- | --- |
| 25 | 5.44 | 0.7 | 6.53 | 0.71 | 6.11 | 0.8 | 2 | 0.2 | 1 | 0.15 |
| 37 | 7.33 | 0.5 | 9.89 | 0.4 | 9.13 | 0.6 | 2 | 0.2 | 1 | 0.15 |
| 50 | 9.22 | 0.6 | 10.22 | 0.63 | 9.47 | 0.65 | 2 | 0.2 | 1 | 0.16 |
| 60 | 10.33 | 0.8 | 11.43 | 0.81 | 10.67 | 0.7 | 2 | 0.3 | 1 | 0.17 |
| 70 | 18 | 0.9 | 21 | 0.72 | 20 | 0.5 | 2 | 0.2 | 1 | 0.22 |
| 80 | 23.25 | 0.7 | 26.33 | 0.83 | 25.33 | 0.72 | 2 | 0.4 | 1 | 0.32 |
| 90 | 26 | 0.5 | 26.33 | 0.73 | 26.33 | 0.83 | 2.11 | 0.5 | 1.11 | 0.43 |

**S4 Fig. Swelling power of native** **and propylated *Dioscorea abyssinica* starches with different degrees of substitution (DS = 0.453, 0.474, 1.710, and 2.842) as a function of temperature.**
