# Supplementary material for: Synthesis, characterization, and in vivo safety evaluation of propylated Dioscorea abyssinica starch
Source: PLoS One. 2022 Nov 28;17(11):e0276965. doi: 10.1371/journal.pone.0276965 (PMC9704604; doi:10.1371/journal.pone.0276965)
Supplement: S5 Fig — (DOCX) [file pone.0276965.s005.docx]

| Temperature (^0^C) | NDAS(DS=00)  (X±SD) | | PDAS(DS=0.453)  (X±SD) | | PDAS(DS=0.474)  (X±SD) | | PDAS(DS=1.710)  (X±SD) | | PDAS(DS=2.842)  (X±SD) | |
| --- | --- | --- | --- | --- | --- | --- | --- | --- | --- | --- |
| 25 | 2.5 | 0.3 | 6.3 | 0.5 | 5.5 | 0.7 | 0 | 0 | 0 | 0 |
| 37 | 3 | 0.2 | 7.1 | 0.4 | 6.4 | 0.6 | 0 | 0 | 0 | 0 |
| 50 | 3.75 | 0.4 | 8.2 | 0.4 | 7.5 | 0.75 | 0 | 0 | 0 | 0 |
| 60 | 5 | 0.5 | 11 | 0.33 | 10 | 0.67 | 0 | 0 | 0 | 0 |
| 70 | 7.5 | 0.33 | 12.5 | 0.5 | 11.5 | 0.5 | 0 | 0 | 0 | 0 |
| 80 | 10 | 0.67 | 15 | 0.67 | 12 | 1 | 2 | 0.1 | 0 | 0 |
| 90 | 12 | 0.5 | 15 | 0.5 | 13 | 1 | 2 | 0.1 | 1 | 0.1 |

**S5 Fig. Relative Solubility of native and propylated *Dioscorea abyssinica* starches** **with different degrees of substitution (DS=0.453, 0.474, 1.710, and 2.842) as a function of temperature.**
