# Supplementary material for: Synthesis, characterization, and in vivo safety evaluation of propylated Dioscorea abyssinica starch
Source: PLoS One. 2022 Nov 28;17(11):e0276965. doi: 10.1371/journal.pone.0276965 (PMC9704604; doi:10.1371/journal.pone.0276965)
Supplement: S6 Fig — (DOCX) [file pone.0276965.s006.docx]

| **Group** | Body weight in grams | | | | |
| --- | --- | --- | --- | --- | --- |
|  | Initial | 1^st^ week | 2^nd^ week | 3^rd^ week | 4^th^ week |
| I (175 mg/Kg) | 188.67 | 192.39 | 196.56 | 201.44 | 208.89 |
|  | 188.38 | 193.14 | 195.89 | 200.99 | 207.66 |
|  | 184.79 | 190.16 | 193.75 | 199.28 | 205.73 |
|  | 167.44 | 173.12 | 177.63 | 181.39 | 187.55 |
|  | 183.67 | 188.17 | 193.14 | 197.81 | 204.32 |
|  | 172.89 | 179.54 | 183.42 | 188.75 | 193.16 |
| II (560 mg/Kg) | 190.15 | 196.03 | 199.73 | 204.11 | 208.44 |
|  | 185.28 | 189.16 | 194.22 | 199.98 | 205.31 |
|  | 157.12 | 161.61 | 166.57 | 171.32 | 177.56 |
|  | 163.77 | 167.33 | 172.91 | 179.25 | 183.01 |
|  | 177.18 | 182.57 | 186.84 | 191.59 | 197.65 |
|  | 169.91 | 173.22 | 178.99 | 183.71 | 189.65 |
| III (1792 mg/Kg) | 169.44 | 173.35 | 178.55 | 182.93 | 189.68 |
|  | 163.28 | 167.64 | 172.34 | 176.37 | 181.97 |
|  | 159.06 | 163.13 | 168.71 | 172.45 | 178.83 |
|  | 175.12 | 179.87 | 183.96 | 191.22 | 195.79 |
|  | 169.26 | 173.73 | 178.03 | 182.73 | 188.88 |
|  | 165.89 | 169.29 | 173.28 | 179.61 | 185.11 |
| IV (Distilled H_2_O) | 161.94 | 166.58 | 168.97 | 173.56 | 181.87 |
|  | 165.86 | 169.40 | 173.16 | 178.34 | 184.21 |
|  | 171.37 | 175.14 | 180.24 | 185.99 | 191.19 |
|  | 176.73 | 180.67 | 184.31 | 189.78 | 196.51 |
|  | 167.55 | 171.33 | 175.81 | 181.21 | 187.36 |
|  | 165.12 | 170.26 | 173.98 | 179.17 | 185.34 |

**S6 Fig. Mean body weight change in rats treated with 175 mg/kg, 560 mg/kg, and 1792 mg/kg of PDS (DS = 2.842) in distilled water as compared to the control group during subacute toxicity study.**
