# Supplementary material for: Synthesis, characterization, and in vivo safety evaluation of propylated Dioscorea abyssinica starch
Source: PLoS One. 2022 Nov 28;17(11):e0276965. doi: 10.1371/journal.pone.0276965 (PMC9704604; doi:10.1371/journal.pone.0276965)
Supplement: S1 Table — (DOCX) [file pone.0276965.s007.docx]

**S1 Table.** **Effect of reaction time, temperature, starch to pyridine ratio and starch to propionic anhydride on propionyl content and degree of substitution of propylated *Dioscorea abyssinica* starch (PDS) (n =3, mean ± SD)**.

| Reaction conditions | Reaction  Time  (h) | Reaction Temperature (^0^C) | Starch:  pyridine  (g/ml) | Starch:  propionic anhydride  (g/ml) | Volume of titrant, HCl, consumed  (ml) | | | Propionyl content (%) | | | Propionyl content (%)  (n =3, mean ±  SD). | DS |
| --- | --- | --- | --- | --- | --- | --- | --- | --- | --- | --- | --- | --- |
| A | 3 | 75 | 1:5.0 | 1:8 | 31.9 | 31.6 | 31:5 | 15.675 | 16.53 | 16.82 | 16.342 ± 0.596 | 0.553 |
| B | 6 | 75 | 1:5.0 | 1:8 | 28.10 | 28.15 | 28:20 | 26.5 | 26.36 | 26,22 | 26.360 ±0.140 | 1.011 |
| C | 12 | 75 | 1:5.0 | 1:8 | 25.8 | 26 | 25.5 | 33.06 | 32.49 | 33.91 | 33.153± 0.715 | 1.397 |
| D | 24 | 75 | 1:5.0 | 1:8 | 19.8 | 19.7 | 19.6 | 50.160 | 50.445 | 50.73 | 50.445 ± 0.285 | 2.842 |
| E | 24 | 50 | 1:5.0 | 1:8 | 30.5 | 30.6 | 30.4 | 19.665 | 19.38 | 19.95 | 19.665± 0.285 | 0.693 |
| F | 24 | 60 | 1:5.0 | 1:8 | 26.2 | 26.4 | 26.5 | 31.92 | 31.35 | 31.065 | 31.445± 0.435 | 1.293 |
| G | 24 | 75 | 1:1.0 | 1:8 | 25 | 25.10 | 25.2 | 35.34 | 35.055 | 34.77 | 35.055± 0.285 | 1.520 |
| H | 24 | 75 | 1:2.5 | 1:8 | 24.8 | 24.6 | 24.4 | 35.96 | 36.48 | 37.05 | 36.497 ± 0.545 | 1.617 |
| I | 24 | 75 | 1:10 | 1:8 | 27.35 | 27.40 | 27.35 | 28.64 | 28.5 | 28.64 | 28.593± 0.081 | 1.130 |
| J | 24 | 75 | 1:5.0 | 1:1 | 32.6 | 32.7 | 32.4 | 13.68 | 13.395 | 14.250 | 13.775 ± 0.435 | 0.453 |
| K | 24 | 75 | 1:5.0 | 1:2 | 32.4 | 32.5 | 32.2 | 14.25 | 13.965 | 14.820 | 14.345 ± 0.435 | 0.474 |
| L | 24 | 75 | 1:5.0 | 1:4 | 23.9 | 24.2 | 24.3 | 38.475 | 37.62 | 37.335 | 37.810 ± 0.593 | 1.710 |
| Blank Sample | | | | | 37.3 | 37.4 | 37.5 | 00 | 00 | 00 | 00 | 00 |

DS: degree of substitution
