# Supplementary material for: Synthesis, characterization, and in vivo safety evaluation of propylated Dioscorea abyssinica starch
Source: PLoS One. 2022 Nov 28;17(11):e0276965. doi: 10.1371/journal.pone.0276965 (PMC9704604; doi:10.1371/journal.pone.0276965)
Supplement: S2 Table — (DOCX) [file pone.0276965.s008.docx]

**S2 Table. Effect of PDS (DS = 2.842) in distilled water on body weight increment of treated and control rats during acute toxicity study.**

| **Group** | Body weight in grams | | |
| --- | --- | --- | --- |
|  | Initial | 1^st^ week | 2^nd^ week |
| I (175 mg/Kg) | 179.76 | 183.93 | 187.65 |
|  | 180.38 | 185.41 | 187.98 |
|  | 177.97 | 183.61 | 186.57 |
|  | 161.44 | 167.21 | 171.36 |
|  | 178.76 | 183.71 | 188.41 |
|  | 168.98 | 175.54 | 179.42 |
| II (560 mg/Kg) | 180.51 | 186.30 | 189.37 |
|  | 176.82 | 180.61 | 185.22 |
|  | 149.12 | 153.16 | 158.75 |
|  | 156.77 | 160.33 | 165.19 |
|  | 171.18 | 176.75 | 180.48 |
|  | 164.19 | 169.22 | 174.99 |
| III (1792 mg/Kg) | 160.44 | 164.53 | 169.55 |
|  | 155.82 | 159.46 | 164.43 |
|  | 152.60 | 156.31 | 161.17 |
|  | 165.12 | 169.78 | 173.69 |
|  | 160.62 | 164.37 | 169.30 |
|  | 159.98 | 163.92 | 167.82 |
| IV (Distilled H_2_O) | 166.49 | 171.58 | 173.97 |
|  | 170.86 | 174.40 | 178.61 |
|  | 161.73 | 165.41 | 170.42 |
|  | 170.37 | 174.67 | 178.13 |
|  | 164.44 | 168.55 | 172.72 |
|  | 160.21 | 165.62 | 168.89 |
