# Supplementary material for: Synthesis, characterization, and in vivo safety evaluation of propylated Dioscorea abyssinica starch
Source: PLoS One. 2022 Nov 28;17(11):e0276965. doi: 10.1371/journal.pone.0276965 (PMC9704604; doi:10.1371/journal.pone.0276965)
Supplement: Attachment — Submitted filename: Response to Reviewers.docx [file pone.0276965.s010.docx]

**PONE-D-22-13047**

**Synthesis, characterization, and *in vivo* safety evaluation of propylated *Dioscorea abyssinica* starch**

Editor comment #1: Please ensure that your manuscript meets PLOS ONE's style requirements, including those for file naming. The PLOS ONE style templates can be found at

https://journals.plos.org/plosone/s/file?id=wjVg/PLOSOne_formatting_sample_main_body.pdf and

https://journals.plos.org/plosone/s/file?id=ba62/PLOSOne_formatting_sample_title_authors_affiliations.pdf

Author response to editor comment #1: The manuscript is now revised based on PLOS ONE style templates

Editor comment #2: Thank you for stating the following financial disclosure:

One of the authors (Y.B.) would like to thank the Center for Innovative Drug Development and Therapeutic Trials for Africa, for sponsoring the work

Please state what role the funders took in the study. If the funders had no role, please state: "The funders had no role in study design, data collection and analysis, decision to publish, or preparation of the manuscript."

If this statement is not correct you must amend it as needed.

Please include this amended Role of Funder statement in your cover letter; we will change the online submission form on your behalf.

Author response to editor comment #2: This statement is incorporated in the cover letter.

Editor comment #3: Please review your reference list to ensure that it is complete and correct. If you have cited papers that have been retracted, please include the rationale for doing so in the manuscript text, or remove these references and replace them with relevant current references. Any changes to the reference list should be mentioned in the rebuttal letter that accompanies your revised manuscript. If you need to cite a retracted article, indicate the article’s retracted status in the References list and also include a citation and full reference for the retraction notice.

Author response to editor comment #3: The reference list is reviewed and the following references (5, 6, 32, 33, 35, 43, 52, 54, 55) are modified by incorporating missing page numbers.

Editor comment #4: Changes are recommended by the reviewers to be incorporated.

Author response to editor comment #4: The manuscript is revised; all the comments given by the reviewers have been accommodated.

**Comments to the Author**

1. Is the manuscript technically sound, and do the data support the conclusions?

The manuscript must describe a technically sound piece of scientific research with data that supports the conclusions. Experiments must have been conducted rigorously, with appropriate controls, replication, and sample sizes. The conclusions must be drawn appropriately based on the data presented.

Reviewer #1: Yes

Reviewer #2: Yes

2. Has the statistical analysis been performed appropriately and rigorously?

Reviewer #1: Yes

Reviewer #2: Yes

3. Have the authors made all data underlying the findings in their manuscript fully available?

The PLOS Data policy requires authors to make all data underlying the findings described in their manuscript fully available without restriction, with rare exception (please refer to the Data Availability Statement in the manuscript PDF file). The data should be provided as part of the manuscript or its supporting information, or deposited to a public repository. For example, in addition to summary statistics, the data points behind means, medians and variance measures should be available. If there are restrictions on publicly sharing data—e.g. participant privacy or use of data from a third party—those must be specified.

Reviewer #1: Yes

Reviewer #2: Yes

4. Is the manuscript presented in an intelligible fashion and written in standard English?

PLOS ONE does not copyedit accepted manuscripts, so the language in submitted articles must be clear, correct, and unambiguous. Any typographical or grammatical errors should be corrected at revision, so please note any specific errors here.

Reviewer #1: Yes

Reviewer #2: Yes

**5. Review Comments to the Author**

Please use the space provided to explain your answers to the questions above. You may also include additional comments for the author, including concerns about dual publication, research ethics, or publication ethics. (Please upload your review as an attachment if it exceeds 20,000 characters)

Reviewer #1: Comments

The manuscript titled “Synthesis, characterization and *in vivo* safety evaluation of propylated Dioscorea abyssinica starch” submitted to plos one was thoroughly reviewed. The study relates to evaluation of propylated Dioscorea abyssinica starch as a pharmaceutical adjuvant. Author propylated the isolated starch and then evaluated it for Crystallinity, morphological structure, thermal behavior, solubility, and safety. Development of natural product-based material is need of the day due to their safety. It is a good study and conducted in a proper way.

Here are some of my observations/ suggestions;

Reviewer #1: Comment 1: Introduction is not properly arranged and needs to be made in line with the theme of manuscript. P#3, First paragraph: Same references are repeated again within the same paragraph, indicating that the text has been taken from a single source.

Author response to Reviewer #1: Comment 1: The statement that is not related with the theme of manuscript is removed.

Reviewer #1: Comment 2: Purity level of the chemicals needs to be mentioned. It should also be stated that they were used as received or further processing was done.

Author response to Reviewer #1: Comment 2: The comment is accommodated in the revised manuscript.

Reviewer #1: Comment 3: It needs to state that in which mode (absorbance or transmittance) was FTIR spectra obtained. The operating software may also be mentioned

Author response to Reviewer #1: Comment 3: FTIR spectra were obtained with transmittance mode and data acquired from FTIR spectrophotometer (PerkinElmer, Spectrum Two DTGS, UK) were plotted in Origin Pro 8.5.1 software. This is stated in the revised version of the manuscript.

Reviewer #1: Comment 4: Tables are not of uniform format.

Author response to Reviewer #1: Comment 4: The format of the tables is made uniform.

Reviewer #1: Comment 5: Conclusion needs to be concise. It has unnecessary text which should be omitted.

Author response to Reviewer #1: Comment 5: The conclusion is made precise.

Reviewer #1: Comment 6: In some references page numbers are missing which should be included.

Author response to Reviewer #1: Comment 6: The missing page numbers of the following references (5, 6, 32, 33, 35, 43, 52, 54, 55) are incorporated.

**Recommendation by Reviewer #1:**

I suggest to accept the manuscript after incorporation of the suggested changes.

Reviewer #2: Comments

This manuscript is clearly written and well organized. The introduction and background are quite reasonable. The manuscript is accepted with minor revision. There are some observations which are submitted as attachment that needs to be addressed by the author.

Reviewer #2: Comment 1: There are multiple spelling and grammatical errors throughout the entire manuscript which needs to be corrected.

Author response to Reviewer #2: Comment 1: The spelling and grammatical errors have been corrected.

Reviewer #2: Comment 2: The objective and need of the study should be clearly mentioned.

Author response to Reviewer #2: Comment 2: The objective of this study is now stated as “The objective of this research work is to develop an effective propylated *Dioscorea abyssinica* starch (PDAS) as a hydrophobic excipient for pharmaceutical applications with a reasonable price.”

Reviewer #2: Comment 3: The source and purity of each chemical used during the study should be added

Author response to Reviewer #2: Comment 3: Comment addressed.

Reviewer #2: Comment 4: List of instruments used during the study should be provided.

Author response to Reviewer #2: Comment 4: The instruments used are specified in the method section. But, the list of instruments is not prepared separately as it is not the requirement of PLOS ONE.

Reviewer #2: Comment 5: Unit of milli liter is mL. In the text, it is mentioned as ml in multiple places throughout the manuscript and needs to be corrected.

Author response to Reviewer #2: Comment 5: Corrected.

Reviewer #2: Comment 6: Methods for Starch extraction from *Dioscorea abyssinica* tuber should be stated completely.

Author response to Reviewer #2: Comment 6: The extraction method is stated completely.

Reviewer #2: Comment 7: Each of the method used during the study should be stated completely and referenced properly.

Author response to Reviewer #2: Comment 7: All the methods are stated completely and properly referenced.

Reviewer #2: Comment 8: The level or degree of decimal given in data in tables and text should be uniform in the table and throughout the manuscript.

Author response to Reviewer #2: Comment 8: The uniformity of significant figures is now maintained.

Reviewer #2: Comment 9: The references of manuscript should be properly arranged as per journal requirement

Author response to Reviewer #2: Comment 9: The references of the manuscript have been prepared as per the Journal’s requirement.

6. PLOS authors have the option to publish the peer review history of their article (what does this mean?). If published, this will include your full peer review and any attached files.

If you choose “no”, your identity will remain anonymous but your review may still be made public

Do you want your identity to be public for this peer review? For information about this choice, including consent withdrawal, please see our Privacy Policy.

Reviewer #1: No

Reviewer #2: Yes: Dr. Farhad Ullah

[NOTE: If reviewer comments were submitted as an attachment file, they will be attached to this email and accessible via the submission site. Please log into your account, locate the manuscript record, and check for the action link "View Attachments". If this link does not appear, there are no attachment files.]

While revising your submission, please upload your figure files to the Preflight Analysis and Conversion Engine (PACE) digital diagnostic tool, https://pacev2.apexcovantage.com/. PACE helps ensure that figures meet PLOS requirements. To use PACE, you must first register as a user. Registration is free. Then, login and navigate to the UPLOAD tab, where you will find detailed instructions on how to use the tool. If you encounter any issues or have any questions when using PACE, please email PLOS at figures@plos.org. Please note that Supporting Information files do not need this step.

*In compliance with data protection regulations, you may request that we remove your personal registration details at any time. (Remove my information/details). Please contact the publication office if you have any questions.*
