# Supplementary material for: Synthesis, characterization, and in vivo safety evaluation of propylated Dioscorea abyssinica starch
Source: PLoS One. 2022 Nov 28;17(11):e0276965. doi: 10.1371/journal.pone.0276965 (PMC9704604; doi:10.1371/journal.pone.0276965)
Supplement: Attachment — Submitted filename: Reviewer comments.docx [file pone.0276965.s009.docx]

**Reviewer Comments:**

This manuscript is clearly written and well organized. The introduction and background are quite reasonable. The manuscript is accepted with minor revision. The following observations need to be addressed before the manuscript is accepted.

a. There are multiple spelling and grammatical errors throughout the entire manuscript which needs to be corrected
b. The objective and need of the study should be clearly mentioned
c. The source and purity of each chemical used during the study should be added
d. List of instruments used during the study should be provided
e. Unit of milli liter is mL. In the text, it is mentioned as ml in multiple places throughout the manuscript and needs to be corrected
f. Methods for Starch extraction from Dioscorea abyssinica tuber should be sated completely
g. Each of the method used during the study should be stated completely and referenced properly
h. The level or degree of decimal given in data in tables and text should be uniform in the table and throughout the manuscript
i. The references of manuscript should be properly arrange as per journal requirement
